# Comparison of non-surgical treatment methods for patients with lumbar spinal stenosis: protocol for a randomized controlled trial

**DOI:** 10.1186/2045-709X-22-19

**Published:** 2014-05-10

**Authors:** Michael Schneider, Carlo Ammendolia, Donald Murphy, Ronald Glick, Sara Piva, Elizabeth Hile, Dana Tudorascu, Sally C Morton

**Affiliations:** 1 School of Health and Rehabilitation Sciences, Department of Physical Therapy, University of Pittsburgh, Pittsburgh, PA, USA; 2 Institute of Health Policy, Management and Evaluation, University of Toronto, Toronto, Ontario, Canada; 3 Clinical Director, Rhode Island Spine Center, Providence, RI, USA; 4 Department of Family Medicine, Alpert Medical School of Brown University, Providence, RI, USA; 5 Departments of Physical Medicine, Family Medicine, and Psychiatry, University of Pittsburgh, Pittsburgh, PA, USA; 6 School of Medicine, Biostatistics, Clinical and Translational Science Institute, University of Pittsburgh, Pittsburgh, PA, USA; 7 Department of Biostatistics, Graduate School of Public Health, University of Pittsburgh, Pittsburgh, PA, USA

**Keywords:** Lumbar spinal stenosis, Spine, Rehabilitation, Physical therapy, Chiropractic, Non-surgical treatment

## Abstract

**Background:**

Lumbar spinal stenosis is the most common reason for spinal surgery in older adults. Previous studies have shown that surgery is effective for severe cases of stenosis, but many patients with mild to moderate symptoms are not surgical candidates. These patients and their providers are seeking effective non-surgical treatment methods to manage their symptoms; yet there is a paucity of comparative effectiveness research in this area. This knowledge gap has hindered the development of clinical practice guidelines for non-surgical treatment approaches for lumbar spinal stenosis.

**Methods/design:**

This study is a prospective randomized controlled clinical trial that will be conducted from November 2013 through October 2016. The sample will consist of 180 older adults (>60 years) who have both an anatomic diagnosis of stenosis confirmed by diagnostic imaging, and signs/symptoms consistent with a clinical diagnosis of lumbar spinal stenosis confirmed by clinical examination. Eligible subjects will be randomized into one of three pragmatic treatment groups: 1) usual medical care; 2) individualized manual therapy and rehabilitative exercise; or 3) community-based group exercise. All subjects will be treated for a 6-week course of care. The primary subjective outcome is the Swiss Spinal Stenosis Questionnaire, a self-reported measure of pain/function. The primary objective outcome is the Self-Paced Walking Test, a measure of walking capacity. The secondary objective outcome will be a measurement of physical activity during activities of daily living, using the SenseWear Armband, a portable device to be worn on the upper arm for one week. The primary analysis will use linear mixed models to compare the main effects of each treatment group on the changes in each outcome measure. Secondary analyses will include a responder analysis by group and an exploratory analysis of potential baseline predictors of treatment outcome.

**Discussion:**

Our study should provide evidence that helps to inform patients and providers about the clinical benefits of three non-surgical approaches to the management of lumbar spinal stenosis symptoms.

**Trial registration:**

ClinicalTrials.gov identifier: NCT01943435

## Introduction

### Background

Nearly 20% of the U.S. population will be 65 years of age or older by the year 2030 and about 34% of them will suffer from some type of osteoarthritis [1]. Lumbar Spinal Stenosis (LSS) is a chronic condition that is caused by degenerative changes in the lumbar spine, which is highly prevalent in the older adult population. Radiographic and clinical data from the Framingham cross-sectional study support a 30% prevalence of degenerative LSS in older adults [2].

The term LSS encompasses a number of conditions that decrease the total area of the central spinal canal, lateral recess, or intervertebral foramen. Although there may be visual evidence of narrowing of these osseous structures on x-ray, MRI or CT scans, symptomatic LSS is characterized by neurogenic claudication and/or radiculopathy [3]. Interestingly, the severity of bony narrowing measured on MRI or CT does not correlate with the severity of patient symptoms [4,5], including the hallmark symptom of the clinical syndrome: significant leg pain that typically worsens with ambulation.

Given the association of LSS symptoms and walking, it is no surprise that individuals with LSS often experience significant functional limitation of walking and associated disability [6]. Ambulation is a key component of overall health, independent living, and fall prevention. Impaired walking is associated with obesity, heart disease, peripheral artery disease, diabetes, and cognitive decline. Both the volume and intensity of walking ability are significantly lower in patients with LSS compared to patients with hip and knee osteoarthritis [7]. Additionally, individuals with LSS have a risk of falling that is comparable to patients with severe knee osteoarthritis [8,9]. The impact of spinal problems on physical function is greater than or similar to the burden on physical function caused by congestive heart failure, cancer, total knee and hip arthroplasty [10]. For all of these reasons, LSS should be considered a high priority condition for clinical research.

Most of the scientific literature on the clinical management of LSS has focused on invasive procedures such as injections and surgery. There is a paucity of literature on usual medical care, chiropractic, physical therapy, and other non-surgical interventions. Patients with recalcitrant symptoms and severe LSS appear to fare better with surgery. The largest clinical trial to date that compared surgical versus non-surgical care for LSS concluded that patients with symptomatic LSS who were treated operatively had greater improvement in pain and function [11]. However, the results from this trial also showed that about a third of patients in the non-operative group had significant improvements in pain and function lasting up to 4 years. This is strong evidence that a subset of LSS patients clearly show improvement without any surgical intervention.

While the most current systematic review of all non-operative treatment options for LSS identified 21 randomized trials, none provided moderate or high quality evidence for any specific treatment option [12]. Instead, low quality evidence from single trials suggests that: 1) epidural injections improve pain and function better than home exercise or in-patient physical therapy; and 2) exercise provides benefit for leg pain and function when compared to no treatment. Another systematic review of epidural injections for the non-surgical treatment of chronic low back pain includes some studies related to treatment of LSS [13]. This systematic review found a total of 10 randomized trials and 11 observational studies; yet only one small randomized trial and two observational studies were specifically related to LSS. In the randomized trial, epidural injections provided significant relief to only 55-65% of the LSS patients.

These reviews of the literature point to a serious lack of research evidence about most non-surgical treatments for LSS. Therefore, this trial is designed to study the comparative effectiveness of three common non-surgical approaches to the management of LSS: 1) usual medical care; 2) clinic-based manual therapy and individualized exercise; and 3) community-based group exercise. We hypothesize that subjects randomized to receive either clinic-based manual therapy/individualized exercise or community-based group exercise will have better outcomes than those randomized to usual medical care. We also hypothesize that the individualized clinic-based individualized approach will lead to better outcomes than the community-based group approach. However, it would be an important finding if we found that both approaches were equally effective due to the potential cost savings of group exercise over clinic-based health care.

This trial is designed to provide clinically relevant evidence that will help to inform the choices confronting clinicians and patients with LSS when faced with the decision about which type of non-surgical treatment to utilize. There is also a paucity of evidence that takes into account what clinical outcomes are important from the patient’s perspective. This is consistent with the goal of all patient centered outcomes research, which is to determine which treatment works best, for whom, and under what circumstances [14].

## Methods/design

### Participants

We will recruit 180 community-dwelling older adults from the Pittsburgh metropolitan area to participate in our clinical trial. Eligibility for participation requires both anatomical and clinical evidence of LSS; potential participants must share diagnostic imaging results (MRI, CT, etc.) showing bony narrowing, while also presenting with signs/symptoms consistent with the clinical syndrome of LSS (neurogenic claudication).

After informed consent, patients who are willing to be randomized and wish to be considered for participation will first undergo a baseline physical examination and screening process to determine if they meet the study’s eligibility criteria.

#### Inclusion criteria

• Age 60 years or older

• Clinical history and diagnostic imaging evidence of lumbar spinal stenosis (LSS)

• Ability to read/write English

• Limitation of walking tolerance related to LSS (neurogenic claudication)

• Ability to attend 2 intervention session per week for 6 weeks

• Ability to engage in mild exercise

• Ability to walk 50 feet without the need for an assistive device

• Willing to be randomized to one of the 3 treatment groups

#### Exclusion criteria

• History of metastatic cancer

• Cauda equina symptoms including saddle paresthesia

• Previous lumbar surgery for LSS or lumbar spinal fusion

• History of severe peripheral artery disease or Ankle Brachial Index < 0.8

• Have been told by a physician that they should not engage in physical exercise

• History of neurologic (e.g., cervical myelopathy, stroke) or neuro-degenerative (e.g., Parkinson’s disease) other than LSS that affects the subject’s ability to walk

• Cannot complete a self-paced walking test (SPWT) for any reason other than symptoms related to LSS (e.g. chest pain, severe hip or knee arthritis, dizziness, etc.)

• Cannot complete SPWT without need for an assistive device

Our inclusion/exclusion criteria are designed to include patients who have a walking intolerance due to neurogenic claudication, and to rule out patients with vascular claudication or other conditions as causes of their limitation of walking. To screen for vascular conditions we will use the Ankle Brachial Index (ABI), which is a simple clinical screening test for peripheral artery disease (PAD). It is performed by using a hand-held Doppler ultrasonic device and sphygmomanometer to record the systolic pressures over the posterior tibial and brachial arteries. The ABI is calculated by dividing the lower extremity by the upper extremity systolic pressure, with the normal ratio being >0.9 [15]. A study of patients with atypical claudication due to either LSS or PAD found good sensitivity/specificity for differentiating PAD in patients with a low ABI [16]. We will exclude patients with an ABI less than 0.8 from participation in our study, for the rationale that they may have a vascular (versus neurogenic) cause of their claudication symptoms.

### Research design

This is a prospective, randomized, controlled single-center trial to compare three non-surgical treatment approaches for patients with LSS. We will recruit 180 community dwelling older adults (age 60 years and older) from the Pittsburgh metropolitan area to participate in this study. The study began in November 2013 and will continue for three years until October 2016. Recruitment strategies will include: direct mailings, advertisements on city busses, informational brochures at the offices of spine surgeons and primary care offices, information articles about our study in senior newspapers and other publications, and informational lectures given at community senior centers and health fairs.

People interested in our study will first be screened by telephone. Potentially eligible persons will be scheduled for a baseline assessment visit for confirmation of eligibility. After obtaining informed consent and confirmation of meeting the inclusion criteria, 180 participants will then be randomized with equal probability to one of three intervention arms: 1) usual medical care; 2) clinic-based individualized exercise and manual therapy; or 3) community-based group exercise. The intervention period will last a total of six weeks in each arm. Outcomes will be measured at two follow-up assessment visits; two weeks after intervention (2 month follow-up) and four months after intervention (6 month follow-up).

### Interventions

Our choice of interventions was informed by reviews of literature combined with input from patients, providers and our community stakeholders. This led us to choose the three intervention arms listed above. All intervention arms will utilize pragmatic protocols that allow for shared decision making with the patient.

Interviews with primary care physicians, physical medicine & rehabilitation (PM&R) physicians, surgeons, as well as community-dwelling older adults indicated that the “usual medical care” approach to the non-surgical management of LSS is a combination of: advice to stay active, use of oral medications to control pain, mood, and/or inflammation, and judicious referral for spinal injections. The most current lumbar spinal stenosis guideline from the North American Spine Society (NASS) suggests that spinal injections provide short term relief of symptoms, but states there is insufficient evidence for making any recommendations about pharmacological treatment [17].

The most current systematic review of non-surgical treatments for LSS concluded there was a paucity of chiropractic and physical therapy clinical trials [12]. The NASS lumbar spinal stenosis guideline concludes that there is insufficient evidence to make any recommendation for or against physical therapy, spinal manipulation, or exercise for the treatment of LSS [17]. Interviews with physical therapists and chiropractors revealed there were common LSS treatment themes, including the use of spinal de-weighting or traction, spine and hip mobilization, and individualized exercise. Two of the co-investigators (CA, DM) have developed clinical protocols that combine both manual therapy and therapeutic exercises into a pragmatic treatment approach for LSS. Two physical therapists and two chiropractors have been trained by these co-investigators to treat our research subjects with these clinical protocols.

We engaged in discussions about LSS with a number of older adults at community centers in the Pittsburgh metropolitan area, as well as patients with LSS in treatment at local chiropractic and physical therapy clinics. They expressed frustration with the ways in which LSS impacted their daily routines, especially with respect to interference with their ability to perform specific physical activities that involved walking or standing. Many of them had joined community-based exercise programs to help them stay physically fit and active. Several health insurance companies have begun to cover the costs of group exercise programs for their Medicare Advantage subscribers. These group exercise and healthy aging classes are aimed at helping older adults become and/or stay physically fit. These programs are offered at varying levels of difficulty, commensurate with the baseline level of fitness activity of the participant. In general, all of these group exercise classes focus on light non-impact aerobics, overall flexibility and low intensity strength training.

The three intervention arms of this study will utilize a pragmatic *effectiveness* design, using protocols that allow for some individualized variation, rather than comparing strict mono-therapy approaches in an *efficacy* design. Our approach is designed to provide more generalizable results by simulating the real-world delivery of these treatment methods. Each of the treatment arms will be conducted in out-patient or community settings. Subjects with a diagnosis of LSS will be randomized to one of 3 possible treatment groups, which are described in detail below. The duration of treatment will be the same for all groups (6 weeks) but the frequency will vary. The medical care group will receive 3 examination visits over 6 weeks; subjects in the other two groups will receive interventions twice per week for a total of 12 visits over 6 weeks. The procedures and protocols within each treatment group were informed by reviews of the literature, as well as by interviews with patients and providers.

#### Group 1: usual medical care

Participants will see a board certified physical medicine physician for a history and examination, after which a determination will be made about a course of treatment that is individualized to the needs of each patient. The physician will be permitted to prescribe any one or combination of the following categories of oral medications, based upon the individual clinical presentation of each subject:

• Non-steroidal anti-inflammatory: such as ibuprofen, celocoxib, diclofenac, or misoprostol

• Adjunctive analgesics: such as acetaminophen, tramadol, pregabalin, or gabapentin

• Anti-depressant agents: such as nortriptyline, duloxetine, sertraline, trazodone, or mirtazapine

The physician will also have the option to refer patients for spinal injections, which can be performed at two cooperating pain clinics in Pittsburgh. The type of spinal injections may be either epidural steroid injections or transforaminal nerve blocks; at the discretion of the pain management physician performing the procedure. In addition to oral medications and/or spinal injections, all subjects would be given advice to stay active, along with basic principles of proper posture and some simple home stretching exercises.

#### Group 2: Clinic-based individualized exercise and manual therapy

Participants in this arm will be treated by a chiropractor or physical therapist, using a pragmatic individualized approach, following the parameters of a clinical protocol that includes the use of the following procedures:

• Light aerobic exercise on a stationary bicycle.

• Distraction-manipulation: a form of manually-assisted segmental lumbar traction manipulation using a specialized treatment table.

• Neural mobilizations: rhythmic stretches of the sciatic and femoral nerves based on the hypothesis that movement of the neural tissues has a mechanical effect on the local neurodynamics [18].

• Hip, sacroiliac and lumbar facet mobilizations: manual mobilizations applied to improve segmental mobility in these joints.

• Soft tissue mobilizations of lumbopelvic and lower extremity muscles: these include manual pressure over taut bands/myofascial trigger points and post-isometric (contract-relax) stretching techniques.

• Home Exercise: patients are advised to use a stationary bike daily, and taught how to perform neural mobilization self-stretches, as well as individualized stretches and core strengthening exercises at home.

#### Group 3: Community-based group exercise

Participants in this arm will attend group exercise classes designed specifically for older adults and taught by a certified physical fitness instructor. These classes will take place in two large community centers in Pittsburgh that offer programs for older adults. Participants can choose between a class that provides chairs for support while seated/standing or another class for people who are comfortable standing without any support. Each class includes of a variety of exercises designed to increase muscular strength, range of movement, and activity for daily living skills. There is a minimal amount of low impact cardiovascular exercise combined with the use of small hand-held weights, elastic tubing and gym balls for gentle resistance exercise. The participants in these classes are monitored carefully for any signs of physical discomfort and are told to go at their own pace, resting whenever necessary. No specific body region is targeted with these exercise classes. The goal is to provide participants with gentle strengthening, balance training, and generalized fitness training for the entire body. Some of the specific exercises included in these classes are: ankle/wrist rotations, partial squats, leg and knee extension/flexion, strength training of the upper arm and chest muscles with elastic tubing, and coordination drills with a gym ball (bouncing, throwing and catching).

### Outcome measures

Our choice of primary outcome measures was informed by input from our community stakeholders and patients with LSS. They stressed the importance of maintaining their independence and their quality of life through improvements in physical function and activity. Suggestions for patient-centered outcomes included improvements in gait, mobility, walking capacity and general physical activity. We have specifically included several of these patient-centered outcome measures, such as measurements of physical activity and walking capacity, into our research design. We will analyze the effectiveness of various treatment options on all of the outcomes that patients have directly informed us are important to them.

The primary self-reported outcome measure will be the Swiss Spinal Stenosis Questionnaire (SSS); a validated 12-item condition-specific instrument for patients with LSS [19]. The SSS is a patient self-reported measure of pain and physical function. Higher scores represent worse symptoms and lower physical function. The 12 items are divided into two subscales; Symptom Severity (further divided into a pain and neuro-ischemic domain) and Physical Function.

The primary performance-based outcome measure will be the Self-Paced Walk Test (SPWT), which is a validated measure of walking capacity in patients with LSS [20]. This test involves having the patient walk comfortably at his/her own pace on a level surface until s/he must rest due to symptoms of back or leg pain. A research assistant follows the patient and measures the total time and distance walked. Patients are not permitted to use an assistive device during the SPWT. We will exclude patients who can walk for a total of 30 minutes without the need to stop, in order to prevent a ceiling effect.

**Figure 1:**
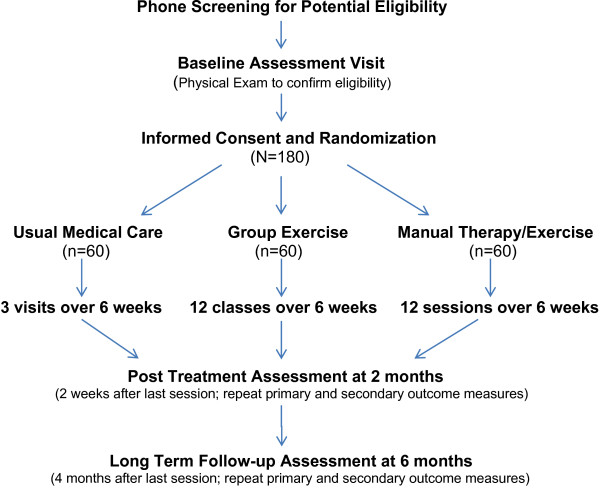
**Study flow diagram.** Primary outcome measures of self-reported pain/function and physical performance are the Swiss Spinal Stenosis questionnaire [19] and the Self-Paced Walking Test [20]. Secondary outcome measure of physical activity is the SenseWear armband (BodyMedia, Pittsburgh, PA) worn for 1 week.

The secondary outcome measure is physical activity measured by a portable activity monitor, the SenseWear Armband (SWA) (Body Media Inc, Pittsburgh, PA). The SWA is a small device that is worn on the upper arm and collects information from multiple sensors: a triaxial accelerometer; heat flux; skin temperature; and galvanic signal [21]. The information is integrated and processed by software using proprietary algorithms utilizing subjects’ demographic characteristics (gender, age, height, and weight) to provide minute-by-minute estimates of physical activity. Subjects will wear the SWA on the back of the right arm 24 hours/day for 7 consecutive days (except during shower or water activities) to obtain 5 complete days of data. Figure 1 provides a diagram of the study flow.

### Additional measurements

Subjects in this study will complete a series of questionnaires and clinical examination procedures at baseline that will provide additional variables. These variables will give us additional data that allows for the option of an exploratory analysis of any individual or cluster of baseline features that might be predictors of treatment response.

The questionnaires will include the following:

• Demographics: including age, gender, race/ethnicity, smoking status, etc.

• Modified Co-Morbidity Disease Index [22]: subjects will be asked to indicate if they have been diagnosed by a physician with any of 19 medical conditions.

• Fall History Form [23]: subjects will be asked about any falls over the past year, and any current fear of falling.

• Oswestry Low Back Pain Disability Index [24]: A validated 10-item instrument for assessing the degree of self-reported impairment of activities of daily living related to low back pain.

• Balance: the Activities-Specific Balance Confidence (ABC) Scale [25] will be administered: a validated questionnaire assessing the patient’s level of self-confidence about not losing their balance while performing 16 upright activities of varying difficulty. Additionally, patients will be asked to provide a global rating of their balance, and to rate change at each outcomes visit.

• Tampa Scale for Kinesiophobia [26]: A validated 11-item self-report measure for assessing fear of movement and injury in low back pain patients.

• Depression [27]: We will use the short-form version of the PROMIS depression scale.

• Treatment Expectation [28]: We will use the 6-item credibility and expectancy questionnaire that was modified for use in patients with chronic low back pain.

Clinical examination procedures will include:

• Lumbar spine, hip and knee joint palpation and ranges of motion.

• Height, weight and blood pressure measurements.

• Ankle Brachial Index: systolic blood pressures at brachial and tibial arteries [15,16].

• Neurological exam: lower extremity deep tendon reflexes, manual muscle testing for strength, Babinski test, assessment of pinprick and vibratory sensation.

• Neurodynamic exam including straight leg raise (sciatic nerve and its roots) and femoral nerve stretches (femoral nerve and its roots).

• Self-Paced Walking test [20]: distance and time walked until test must be self-terminated due to symptoms, or 30 minutes, whichever occurs first.

• Balance and Mobility: A modified version of the Short Physical Performance Battery [29] will be administered, in which two of the original subscales (4-meter walking speed and timed chair stands) are combined with an expanded timed standing balance assessment.

### Statistical analysis

#### Outcome measures and primary analysis

The primary outcome variables are the Swiss Spinal Stenosis (SSS) score and Self-Paced Walking Test (SPWT) measured at 2 months (2 weeks after intervention); both will be analyzed similarly. We will also assess these outcomes at 6 months. The outcomes and all baseline characteristics will be summarized with descriptive statistics, separated by treatment group. Analysis of variance (ANOVA) will be used to assess the unadjusted associations between each outcome variable and treatment group membership. A multivariable linear regression model will be used to assess the significance of treatment for each group while adjusting for baseline variables that are at least marginally significant univariately (p < 0.10) and other variables that are selected a-priori for clinical significance (e.g. age). All analysis for treatment group comparisons will use an intention-to-treat approach.

#### Power analysis

A 30% difference between groups on the primary outcome measures will be considered a clinically important difference. A sample size of 150 subjects equates to over 90% power to detect a difference of 9.9 points on the SSS score (with a SD of 6.1 based on preliminary data). In addition, this sample size also yields sufficient power to fit a regression model that detects a statistical difference in the proportion of variability explained; more specifically, a sample size of 150 achieves 81% power to detect an R-square of 5% attributed to two independent variables (representing the two degrees of freedom in the three levels of treatment) and assuming the control variables account for an additional 20% of the variability. We will recruit an additional 30 subjects to account for an anticipated drop-out rate of 15%. This gives us a total sample size of 180 subjects (n = 60 per group).

#### Secondary analysis

We will also perform a responder analysis using dichotomous outcomes, consistent with the recommendations published by the Initiative on Methods, Measurement, and Pain Assessment in Clinical Trials (IMMPACT) [30,31]. Our responder analyses will compare the percentages of subjects who achieve meaningful outcomes between treatment groups on SSS score and SPWT (distance walked). Per IMMPACT recommendations, subjects who achieve at least 30% and 50% decreases in outcome measures are considered to show “moderate improvement” and “substantial improvement”, respectively. Differences in the proportion of responders versus non-responders between the groups will be assessed using Chi-square tests. Multiple logistic regression models will be used to assess the association of each of these dichotomized outcomes (>30% followed by >50%) by group, adjusted for covariates. The covariates will be chosen based upon univariate logistic regression (p < 0.10) and clinical relevance.

#### Missing data

If a substantial number of subjects are found to have missing data, we will first determine the causes of why data are missing. These possibilities could be missing completely at random, missing at random, or not missing at random [32]. We will compare characteristics between subjects that have observed data at follow up with those that have missing values. In the case where data is missing at random or completely at random, multiple imputation will be used to substitute for the missing values using a pre-specified model. Once the imputed datasets have been created (we will use M = 5 imputations) the proposed analysis will be performed on each of the new datasets and the estimates of interest will be combined [33].

In the case of not missing at random, the missing data mechanism must be modeled in order to obtain unbiased parameter estimates. The missing not at random assumes the missingness is directly related to the value of the missing data. In that case, methods specifically designed to handle the missing not at random mechanism will be used such as Pattern Mixture Models (PMM) [34]. With the PMM, we can simultaneously model whether a subject is a completer (has the data at both time points) versus a non-completer (does not have the data at both time points). We would include an indicator variable for the non-completers as a predictor in the regression model of our outcome measure and examine its interaction with the study covariates. The results of all missing data analysis will be presented in the primary paper for our study. Statistical analysis will be performed using SAS 9.3 (SAS Institute Inc., Cary, NC, USA).

#### Randomization and blinding

Randomization will be performed by the research coordinator after the baseline screening examination using a computerized randomization algorithm. We will use an adaptive allocation algorithm to balance the three groups on important baseline variables. The randomization scheme is based on a minimization algorithm proposed by Stigsby and Taves [35]. The approach is to balance on multiple baseline prognostic factors using a rank-based method. The balancing baseline variables used will be: Swiss Spinal Stenosis Score; Self-Paced Walking Test (distance walked); Age; and Credibility Expectancy Questionnaire score.

Single blinding will be achieved by having an independent physical therapist perform all baseline exams and post-intervention reassessments. The examining therapist will not be treating any of the subjects or aware of their group assignments. Blinding of the treating clinicians and subjects is not possible because they will obviously be aware of the treatment arm to which they have been assigned.

#### Ethical considerations

This study will be conducted in accordance with the declaration of Helsinki and all ethics regulations, policies and guidelines of the National Institutes of Health. The Institutional Review Board of the University of Pittsburgh has reviewed and approved this study (PRO12120422). Written informed consent will be obtained from all patients included in the study. Patients will be informed that they are free to leave the study, without explanation and without any negative consequences on their future treatment. Every precaution will be taken to protect the privacy of research subjects and the confidentiality of their personal information. All personal patient details will be rendered anonymous before data entry, by referring to all patient records and data only by their assigned research number. There are no known additional risks associated with patient participation in the study, other than the normal risks associated with these common treatments.

## Discussion

Patients with severe symptoms will often require surgical decompression; LSS is the most frequent indication for spinal surgery in the U.S. for patients over the age of 65 years [36]. Clearly, not all LSS patients require surgery and therefore we are only recruiting patients with mild to moderate symptoms who are not surgical candidates. This leads to some important research questions:

• Among the commonly used non-surgical approaches to treat LSS, which ones are most effective at reducing pain and improving walking capacity?

• How much impact does successful treatment have on improving physical activity?

• What are the benefits/risks associated with the various non-surgical treatments for LSS?

• Are there baseline physical examination and case history findings that are predictors of treatment response?

As noted in the introduction section of this article, there is a serious evidence gap about the effectiveness of these various non-surgical treatment approaches. The seriousness of this gap is highlighted by these recommendations from the evidence-based clinical guideline for LSS recently published by the North American Spine Society [17]:

• There is insufficient evidence to make a recommendation for or against the use of pharmacological treatment in the management of LSS.

• There is insufficient evidence to make a recommendation for or against the use of physical therapy or exercise as stand-alone treatments for degenerative LSS.

• There is insufficient evidence to make a recommendation for or against the use of spinal manipulation for the treatment of LSS.

There is essentially insufficient evidence for every common non-surgical treatment approach except epidural steroid injections; and these injections only have one randomized trial showing evidence of modest effectiveness. This puts patients and providers in an uncomfortable position of uncertainty about which approach to non-surgical management is best for which patient. This study is designed to provide evidence to help remove some of this clinical uncertainty experienced by providers who must recommend these treatments. It is also designed to measure outcomes that are important to patients, including measures of physical activity and walking capacity; two of the most patient-centered outcomes reported to us by patients with LSS and their care-givers.

## Conclusions

This study will be one of the first of its kind: comparing the clinical effectiveness of three common non-surgical approaches to the management of patients with LSS. We have also designed this study with input from patients with LSS and other community stakeholders. Our research design is also innovative because all treatment groups involve “real-life” management strategies, using pragmatic protocols that can be adapted to individual patients’ needs.

## Abbreviations

ABI: Ankle-brachial index; LSS: Lumbar spinal stenosis; PAD: Peripheral artery disease; PM&R: Physical medicine and rehabilitation; SPWT: Self-paced walking test; SSS: Swiss spinal stenosis questionnaire.

## Competing interests

The authors declare that they have no competing interests.

## Authors’ contributions

MS initiated the study and had the primary responsibility of writing the grant application and applying for funding. CA and DM are clinical chiropractic experts in the non-surgical management of LSS and developed the clinical treatment protocols for the manual therapy/exercise group. SP and EH are academic physical therapy experts who have clinical and research experience working with older adults, including the use of the SenseWear device to monitor physical activity and the assessment of mobility and falls. They helped to select the outcome measures and refine the exercise protocols. They will also lead the analyses of physical activity, mobility, and falls. RG is a clinical medical expert who is board certified in physical medicine and rehabilitation. He was involved with the design of the usual medical care arm of the study. DT and SM are biostatisticians who performed the power analysis, programmed the randomization algorithm, and will analyze all data at the end of the study. All authors read, revised, and approved this final manuscript, as well as the original grant proposal.
